# Mapping and selection of downy mildew resistance in spinach cv. whale by low coverage whole genome sequencing

**DOI:** 10.3389/fpls.2022.1012923

**Published:** 2022-10-06

**Authors:** Gehendra Bhattarai, Dotun Olaoye, Beiquan Mou, James C. Correll, Ainong Shi

**Affiliations:** ^1^ Department of Horticulture, University of Arkansas, Fayetteville, AR, United States; ^2^ Crop Improvement and Protection Research Unit, United States Department of Agriculture, Agricultural Research Service, Salinas, CA, United States; ^3^ Department of Plant Pathology, University of Arkansas, Fayetteville, AR, United States

**Keywords:** spinach, downy mildew, oomycete, disease resistance, mapping, GWAS, candidate gene, breeding

## Abstract

Spinach (*Spinacia oleracea*) is a popular leafy vegetable crop and commercial production is centered in California and Arizona in the US. The oomycete *Peronospora effusa* causes the most important disease in spinach, downy mildew. A total of nineteen races of *P. effusa* are known, with more than 15 documented in the last three decades, and the regular emergence of new races is continually overcoming the genetic resistance to the pathogen. This study aimed to finely map the downy mildew resistance locus *RPF3* in spinach, identify single nucleotide polymorphism (SNP) markers associated with the resistance, refine the candidate genes responsible for the resistance, and evaluate the prediction performance using multiple machine learning genomic prediction (GP) methods. Segregating progeny population developed from a cross of resistant cultivar Whale and susceptible cultivar Viroflay to race 5 of *P. effusa* was inoculated under greenhouse conditions to determine downy mildew disease response across the panel. The progeny panel and the parents were resequenced at low coverage (1x) to identify genome wide SNP markers. Association analysis was performed using disease response phenotype data and SNP markers in TASSEL, GAPIT, and GENESIS programs and mapped the race 5 resistance loci (*RPF3*) to 1.25 and 2.73 Mb of Monoe-Viroflay chromosome 3 with the associated SNP in the 1.25 Mb region was 0.9 Kb from the NBS-LRR gene SOV3g001250. The *RPF3* locus in the 1.22-1.23 Mb region of Sp75 chromosome 3 is 2.41-3.65 Kb from the gene Spo12821 annotated as NBS-LRR disease resistance protein. This study extended our understanding of the genetic basis of downy mildew resistance in spinach cultivar Whale and mapped the *RPF3* resistance loci close to the NBS-LRR gene providing a target to pursue functional validation. Three SNP markers efficiently selected resistance based on multiple genomic selection (GS) models. The results from this study have added new genomic resources, generated an informed basis of the *RPF3* locus resistant to spinach downy mildew pathogen, and developed markers and prediction methods to select resistant lines.

## Introduction

Spinach (*Spinacia oleracea* L.) is an important cool-season leafy vegetable crop. The US annually cultivates spinach on 58,000 acres with a value of 500 million dollars (USDA NASS, 2020) and ranks second in production after China. More than 90% of spinach in the US is produced during the mild-cool season in California and Arizona, providing a year-round fresh supply. Spinach is a diploid crop with six pairs of chromosomes (2n = 2x = 12) and is primarily a dioecious crop comprising separate male and female plants. Spinach is nutritious and an excellent source of health-promoting compounds and nutrients (Morelock and Correll, 2007).

Downy mildew, caused by the obligate oomycete *Peronospora effusa*, is the most economically important disease of spinach, making the produce unmarketable after infection. Breeding for resistance to downy mildew pathogen is the primary objective of all spinach breeding programs (Morelock and Correll, 2007; Bhattarai and Shi, 2021). Nineteen unique races of *P. effusa* have been documented (Feng et al., 2014; Feng et al., 2018b; Plantum, 2021) in spinach, of which sixteen were reported in the last three decades. New races of *P. effusa* have continually overcome newly deployed genetic resistance making downy mildew a major challenge for sustainable spinach production. Significant increase in the production area in recent decades and planting in a higher density, year-round production, and use of resistant cultivars with narrow genetic backgrounds increase selection pressure, and increased organic production provides favors *P. effusa* growth and multiplication, and all these phenomena promote emergengence of new pathogen races. Recent studies have reported asexual genetic variation, the presence of opposite mating types among California isolates (Dhillon et al., 2020), and sexual recombination within the *P. effusa* population (Lyon et al., 2016; Kandel et al., 2019), all of which might contribute to the emergence of new races.

Most spinach cultivars resistant to downy mildew were bred using single-gene resistance against the various races of *P. effusa.* Different *RPF* loci (Resistance to *Peronospora farinosa*) have been hypothesized to provide resistance to races of *P. effusa* (Correll et al., 2011). Commercial hybrid cultivars are developed using a single or combination of a few *RPF* genes from two parents. Genetic investigation and characterization of the resistance sources and identifying molecular markers linked to the resistant genes will facilitate R-gene pyramiding and breeding new resistant cultivars. The *RPF1* locus was mapped to chromosome 3 and a codominant marker DM1 was identified at 1.7 cM from the *RPF1* locus (Irish et al., 2008). Later, the *P. effusa* resistance loci *RPF1*, *RPF2*, and *RPF3* were mapped to a 1.5 Mb region of chromosome 3 (Feng et al., 2015; Feng et al., 2018b), *RPF1* locus was further narrowed to a 0.89 Mb region between 0.34-1.23 Mb (She et al., 2018), and candidate genes predicted in providing resistance to *P. effusa* based on Sp75 assembly were reported. The *P. effusa* resistance region was finely mapped using genotyping by sequencing (GBS) based SNP markers in segregating populations inoculated with *P. effusa* race 13 to 0.84 Mb (Bhattarai et al., 2020b) and race 16 to 0.57 Mb (Bhattarai et al., 2021) region of Sp75 assembly. Recently, the resistance to downy mildew pathogen from the germplasm panel was mapped to the 0.3-1.5 Mb region of the Monoe-Viroflay assembly, containing six NBS-LRR proteins encoding genes (Cai et al., 2021). However, the downy mildew disease resistance loci in spinach have only been molecularly tagged, DNA markers have been developed, but the resistance regulating genes have not been isolated, and functions are still unknown.

Demand for spinach in the US is continually increasing and organic production comprises around 50% of the total production. The utilization of host genetic resistance in developing new resistant cultivars is the most promising disease management approach in crops, particularly in organic production, where the use of resistant cultivars is the only viable disease management option. Identifying additional resistance sources against races of *P. effusa* and expanding the understanding of the mechanism of genetic resistance could provide new options and effective molecular selection tools to improve the durability of resistance. Genetic mapping of the resistance sources and identification of gene-based markers are expected to facilitate R-gene pyramiding. In addition to the major R gene-based disease resistance strategy, the identification of susceptibility genes (S-genes) are being approached that may open alternative avenues to develop resistant cultivars by loss-of-function of the S-genes (Bhattarai et al., 2020b; Ribera et al., 2020). The use of the susceptibility gene in providing effective resistance are reported and established in other crop pathogen system (Bai et al., 2008; Pavan et al., 2011; Pessina et al., 2016; Wang et al., 2016a) and may be effective in providing durable and non-race-specific resistance in spinach. Regular overcoming of resistance genes by new races has urged a better understanding of the molecular host-pathogen battle at the molecular level to support holistic disease management options and using host genetic resistance.

Decreasing sequencing and genotyping costs in the past decade allowed the adoption of quantitative trait loci (QTL ) mapping and genome wide association study (GWAS) for small breeding programs to identify, map, and characterizes genomic regions controlling the phenotypic expression. Genetic resistance to downy mildew pathogens has been extensively studied in many crops (Bachlava et al., 2011; Parra et al., 2016; Wang et al., 2016b; Pyne et al., 2017). Biparental QTL mapping requires the development of progeny population segregating for the trait to detect QTLs, while GWAS allows mapping the trait and identification of genetic variants associated with the trait in diverse germplasm or multi-parent progeny population. Seevral traits in plants and animals have been mapped using the GWAS approach, including resistance to downy mildew pathogen in spinach (Bhattarai et al., 2020b; Bhattarai et al., 2021; Cai et al., 2021). Genomic selection (GS) predicts the breeding value of complex traits of the test population by assessing the effect of genome wide markers, facilitating the selection of superior genotypes without phenotyping and field tests, and accelerating breeding cycles (Meuwissen et al., 2001; Heffner et al., 2009; Bernardo, 2010; Jannink et al., 2010). In the past two decades, GS has been reported in several horticultural and agronomic crops for qualitative and quantitative traits in biparental, multiparent, and natural populations (Lorenzana and Bernardo, 2009; Heffner et al., 2011; Gezan et al., 2017; Poudel et al., 2019; Islam et al., 2020; Sehgal et al., 2020), including resistance to downy mildew and white rust pathogen in spinach (Bhattarai et al., 2022; Shi et al., 2022). Several parametric (rrBLUP-ridge regression BLUP, Bayes A, Bayes B, Bayesian LASSO) and nonparametric (RKHS-Reproducing Kernel Hilbert Space, RF-Random Forest, SVM-Support Vector Machine) models are optimized to increase prediction accuracy in plant and animal breeding programs. The development of reduced representation sequencing, low coverage resequencing, and targeted sequencing in the past decade allowed generation of genotypic datasets at a reasonable cost for plant breeding programs offering options to perform GWAS and GS to improve selection methods and increase selection accuracy. We employed the low coverage whole genome resequencing approach to sequence the population and get genotype data in this study, and this approach are described for many other crops in trait dissection in diverse and bi-parental populations (Gao et al., 2013; Bayer et al., 2015; Hu et al., 2018; Malmberg et al., 2018).

The spinach cultivar Whale is known to contain the *RPF3* allele and provide resistance to *P. effusa* race 1, 3, 5, 8-9, 11-12,14, 16, and 19 (Feng et al., 2014; Feng et al., 2018b; Bhattarai et al., 2020a; Plantum, 2021), and is widely used as a differential set to discriminate the races and isolates of downy mildew pathogen. This study used the GWAS method to finely map genomic regions controlling resistance to downy mildew pathogen race 5 from an F2 population segregating for *RPF3* locus from the differential cultivar Whale. The specific objectives of this study were to identify the SNP markers associated with the resistance to the downy mildew pathogen, identify and refine the candidate genes involved in resistance, and evaluate machine learning tools in predicting resistance.

## Materials and methods

### Plant materials and populations

The cultivar Whale contains *RPF3* locus and is resistant to race 5 of *P. effusa*. A segregating F2 population was developed by crossing Whale and Virfolay (susceptible cultivar) and a cross between resultant F1 males and female plants. Seeds were harvested from each of the female plants representing a progeny population. Initially, 10-20 F2 seeds and parent lines were evaluated for disease response upon inoculation with *P. effusa* race 5. After an initial screening, two progeny populations (VW #10 and VW #3), comprising 137 and 251 seedlings, were inoculated with *P. effusa* race 5 at the Rosen Alternative Pest Control Center, University of Arkansas. Parental cultivars and the differentials, including NIL1, NIL3, and Viroflay, were included in the disease screening as controls. Seeds were sown in 25 x 50-cm plastic trays filled with potting soil (Sun Gro Horticulture, Canada). Each plant tray contained ten rows, and 10-15 seeds per row were planted. After germination, 6-8 plants were kept per row and were labeled using a plant tag. Plants were grown in the greenhouse (25°C) for two weeks, watered daily, and fertilized weekly using Miracle-Gro^®^ All Purpose Plant Food.

### Downy mildew inoculation and disease screening

**Figure 1:**
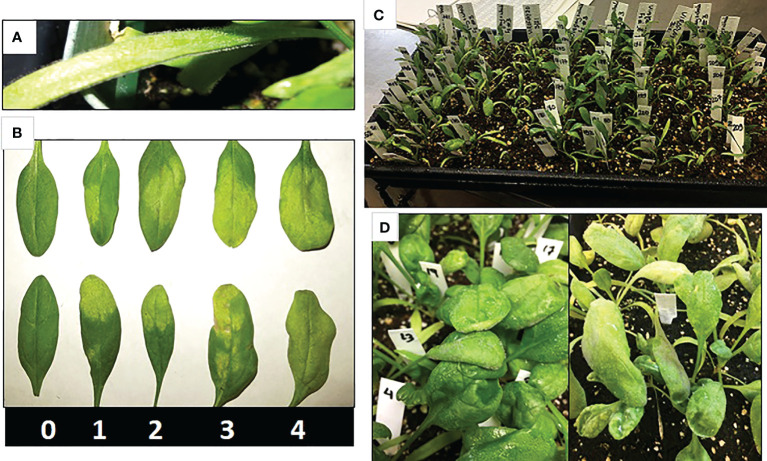
Signs and symptoms in spinach plant infested with downy mildew pathogen. Disease reactions of each plant are rated seven days post inoculation (dpi) based on the presence and absence of sporulation on cotyledons **(A)** and true leaves on a scale of 0 to 4, with 0 = no sporulation; 1 = up to 25% leaf area with sporulation; 2 = 26 to 50% leaf area with sporulation; 3 = 51 to 75% leaf area with sporulation; and 4 = 76 to 100% leaf area with sporulation **(B)**. Progeny populations are inoculated in a tray **(C)** and are scored for disease reaction **(D)** based on sporulation in leaf and cotyledons.

Before inoculation, one leaf from each labeled seedling was excised and stored for DNA extraction. Seedlings in trays were inoculated following the standard whole plant inoculation method (Feng et al., 2018b; Bhattarai et al., 2020b). Briefly, the inoculation assay involves growing plants for two weeks in the greenhouse. Fresh inoculums were prepared every week on susceptible cultivar Viroflay, conidia were washed off from the infected leaves in the cold (4°C) distilled water, and spore suspension diluted to 10^5^ spores per ml was used to inoculate using a Badger basic spray gun (model 250) until the leaves were wet. Inoculated plants in trays were incubated in a dew chamber (18°C) for 24 h in the dark, moved to a growth chamber (18°C, 12 h dark-light cycle) for five days, and finally returned to the dew chamber (18°C) for 24 h to induce sporulation. The disease reactions of each plant were rated seven days post inoculation (dpi) based on the presence and absence of sporulation on cotyledons and true leaves on a scale of 0 to 4, with 0 = no sporulation; 1 = up to 25% leaf area with sporulation; 2 = 26 to 50% leaf area with sporulation; 3 = 51 to 75% leaf area with sporulation; and 4 = 76 to 100% leaf area with sporulation (**Figure 1**). A plant was scored qualitatively as “resistant” if both cotyledons and leaves showed no sporulation, otherwise scored as “susceptible.” Plants were re-inoculated and kept in the growth chamber and dew chamber for an additional week, and the disease was re-scored for a second time to minimize the phenotyping error as downy mildew disease was evaluated based on the reaction of a single plant. In addition, resistant plant in the vicinity of diseased plants was genotyped, while multiple resistant plants in the tray-row were excluded to help increase the confidence of single plant-based disease response.

### Sequencing and marker discovery

Genomic DNA was extracted with Omega MagBind Plant DNA DS kit (Omega Bio-tek Inc., Norcross, GA, USA) in an automated KingFisher Flex extraction system (Thermo Fisher Scientific, Waltham, MA, USA). Extracted DNA was quantified using a Qubit Fluorometer, sample integrity was tested on 1% agarose gel electrophoresis, and DNA meeting the requirements were submitted for sequencing at the Texas A&M genomics facility.

The whole genome resequencing (WGR) was pursued to generate around 1 Gb sequence reads per sample, approximating 1x genome coverage. Variants were called by mapping the sequence reads to the Monoe-Viroflay reference genome (Cai et al., 2021) using the Illumina Dynamic Read Analysis for GENomics (DRAGEN) pipeline (v 3.8.4). SNP variants were initially filtered using BCFtools (Li, 2011) for a minimum coverage depth of 3, minimum genotype quality (GQ) < 9, minor allele frequency (MAF) < 0.05, and missing rate > 75% were removed. The datasets were imputed using Beagle 4.1 (Browning and Browning, 2016), and imputed calls with genotype probability < 0.9 were removed.

Next, SNPs from six chromosomes were extracted and further filtered using BCFtools (Li, 2011) to remove monomorphic SNPs, keep only biallelic SNPs, and remove indels and SNPs within ten bp of indels. The SNP data were filtered for over 25% of missing calls using BCFtools, heterozygosity > 30%, and allele < 5%. Finally, removing SNP with no polymorphism between Whale and Viroflay retained a filtered 8,189 high-quality SNPs for downstream analysis. Similar parameters were used to map the sequencing reads to the SP75 reference assembly (Xu et al., 2017), and filtering resulted in a set of 13,476 SNPs that were also used for GWAS analysis.

### Population structure and clustering

Genetic diversity and principal component analysis (PCA) were performed using the 8189 SNPs in GAPIT3 (Lipka et al., 2012; Wang and Zhang, 2021) programs by setting PCA and NJ tree =2. An unweighted neighbor-joining (NJ) tree and PCA plot were drawn in GAPIT3. The PCA was also performed in TASSEL (Bradbury et al., 2007) and GENESIS (Gogarten et al., 2019) programs to use as a covariate for GWAS.

### Genome wide association analysis for *RPF3* locus

Initial GWAS analysis was performed using single marker regression (SMR), general linear model (GLM) with two PCA matrices, and mixed linear model (MLM), including in-built kinship and PCA matrices in TASSEL 5.2.82 (Bradbury et al., 2007). A second association analysis was performed using the GLM, MLM, and MLMM models in the GAPIT3 R package (Lipka et al., 2012; Wang and Zhang, 2021). A final GWAS was run using the logistic mixed model (LMM) by incorporating inbuilt PCAs and kinship matrices in the GENESIS R Bioconductor package (Gogarten et al., 2019). The TASSEL and GAPIT3 models are more suitable for quantitative phenotype, while the GENESIS model explicitly handles the qualitative phenotype of two classes with case/presence and control/absence response. Downy mildew disease score was changed to 1 for resistant and 9 for the susceptible response as phenotype dataset in TASSEL and GAPIT3, while the score of 0 for resistant and 1 for susceptible was in the GENESIS program. Manhattan plots and QQplots for all association models were drawn using the CMplot package in R. Bonferroni significance threshold (0.05/n) of 5.21 is suggested to control false-positives, but we only considered LOD value > 6.0 to report marker associations in this study. In addition, genome wide SNP data generated by mapping to SP75 reference assembly (Xu et al., 2017) was used for GWAS analysis using all ten models.

### Candidate gene identification

Significantly associated SNPs identified from multiple association models and programs were used to search for candidate genes up to 20 Kb on either side of the Monoe-Virofaly genome assembly. Genes near the peak associated SNPs were examined for annotated functions. Genes predicted to provide disease resistance against plant pathogens were considered potential candidate genes, and their predicted functions were reported. In the same way, candidate genes were searched for GWAS associated SNPs based on the Sp75 assembly (Xu et al., 2017).

### Genomic selection

Prediction performance of resistance to downy mildew pathogen in this population was explored using five different machine learning methods: ridge regression best linear unbiased prediction (rrBLUP), Bayesian models Bayes B, Bayesian LASSO, and Bayesian ridge regression (BRR), and support vector machine (SVM). The SVM model was included as it is more suitable for non-linear data fitting, while other models are more suitable for linear functions. The rrBLUP was fitted using the rrBLUP R package (Endelman, 2011), the Bayesian models using the BGLR R package with 3000 iterations and 1000 burn-in (Pérez and De Los Campos, 2014), and the SVM model implemented in a kernlab R package (Karatzoglou et al., 2004).

GP was performed following a five-fold cross-validation scheme where individuals are randomly assigned into five groups, retaining four groups as the training set (80% of individuals) and the remaining fifth group (20% of individuals) serving as the validation set to predict genomic estimated breeding values (GEBV). The cross-validations were replicated 100 times and prediction accuracy (PA) was determined by averaging the Pearson correlation coefficient (r) between predicted GEBV values obtained from five-fold cross-validations and observed phenotype values in the validation set. Four sets of marker datasets were evaluated with each of the five GP models to compare prediction accuracies among full marker data sets and a smaller number of trait-associated marker sets and to determine the optimum number of markers to obtain high PA for resistance to downy mildew pathogen. The first marker set was the full set of 8,189 SNPs based on the Monoe-Viroflay assembly used for the GWAS analysis. The second set contained 215 SNP markers associated with resistance with LOD ≥ 3.0 in the SMR model in TASSEL. The third set contained 20 SNP with LOD > 6 in all three programs (TASSEL, GENESIS, and GAPIT3), and the fourth set contained three selected significant SNPs.

## Results

### Resistance response to *P. effusa* race 5

**Figure 2:**
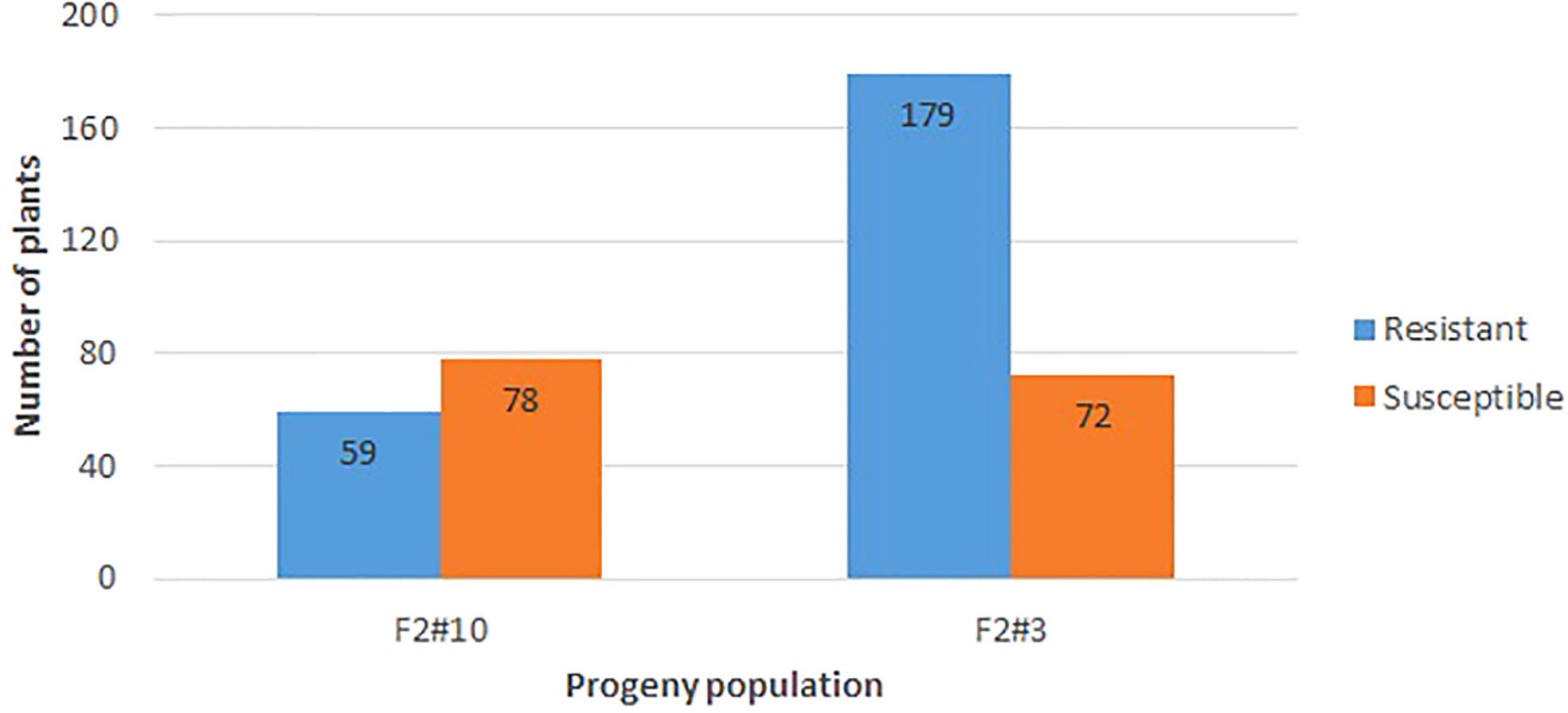
Disease response of the F2 progeny segregating from a cross of Viroflay x Whale inoculated with race 5 of *P. effusa* in the greenhouse condition. Whale is the resistant parent, and Viroflay is susceptible to all known races of downy mildew pathogens (*P. effusa*). The number of resistant and susceptible seedlings in all tested progeny populations is noted.

The resistant parent Whale and susceptible parent Viroflay showed expected responses with race 5 of *P. effusa* in all greenhouse inoculation experiments. The F2 progeny populations following the greenhouse inoculation show segregation for resistance to downy mildew pathogen (**Figures 1C, D**), and the disease response of the progeny panel is presented (**Figure 2**). Of the 137 seedlings of VW #10, 59 were resistant, and 78 were susceptible, showed an excess of susceptible seedlings, and did not fit the expected 3:1 ratio but fit the 1:1 segregation ratio (χ^2^ = 2.64, *P*= 0.10). However, of the 251 seedlings of the second population, VW #3, 179 were resistant and 72 susceptible and fit the 3:1 segregating ratio (χ^2^ = 1.82, *P* = 0.18). The progeny populations VW #3 segregating for *RPF3* locus from Whale fitted to a 3:1 expected ratio for a single dominant gene; thus, 190 seedlings of this progeny plus the two parents (Whale and Viroflay) were selected for sequencing and to investigate the genetic resistance.

### Sequencing and SNP discovery

In total, 183.57 Gb data containing 1,223.82 million raw reads were generated from the Illumina sequencing with an average of 6.37 million reads/sample and average genome coverage of 1.07x. High-quality bases (> Q30) and excluding duplicates and clipped bases were aligned to the spinach reference genome (Cai et al., 2021) using the Illumina Dynamic Read Analysis for GENomics (DRAGEN) pipeline (v 3.8.4), resulting in 127.5 GB of aligned data containing 857.08 million reads. The SNP variants were called by enabling the variant caller option in DRAGEN. Raw SNP dataset was filtered for minimum coverage depth of 3 (DP 3), minimum genotype quality 9 (GQ 9) at SNP variants, minor allele frequency (MAF) of 0.05, and SNP with missing rates > 75% using BCFtools (Li, 2011). This filtering resulted in 617,998 SNP with missing rates of 68.01%. A total of 613,013 SNPs in six spinach chromosomes were retained following Beagle imputation. The SNP dataset was further filtered for monomorphic SNPs, keeping only biallelic SNPs, missing > 25%, heterozygosity > 30%, and MAF <5%, and removing identical genotype calls in Viroflay and Whale, retaining a final filtered 8,189 SNPs in six spinach chromosomes. Additionally, SNPs calls using the SP75 reference assembly using the same parameters used for the Monoe-Viroflay discussed above were used for GWAS analysis in all ten models. The associated SNPs identified in this study with the SNP dataset based on the two reference assemblies were compared.

### Population structure and principal component analysis

Spinach lines in this panel were differentiated into two main subpopulations in the NJ dendrogram and PCA plot generated by the GAPIT3 program (**Supplementary Figure 1**). The first two internally computed principal components were used as fixed effect covariates in all three GWAS programs, TASSEL, GAPIT3, and GENESIS.

### GWAS of *RFP3* resistance locus

**Figure 3:**
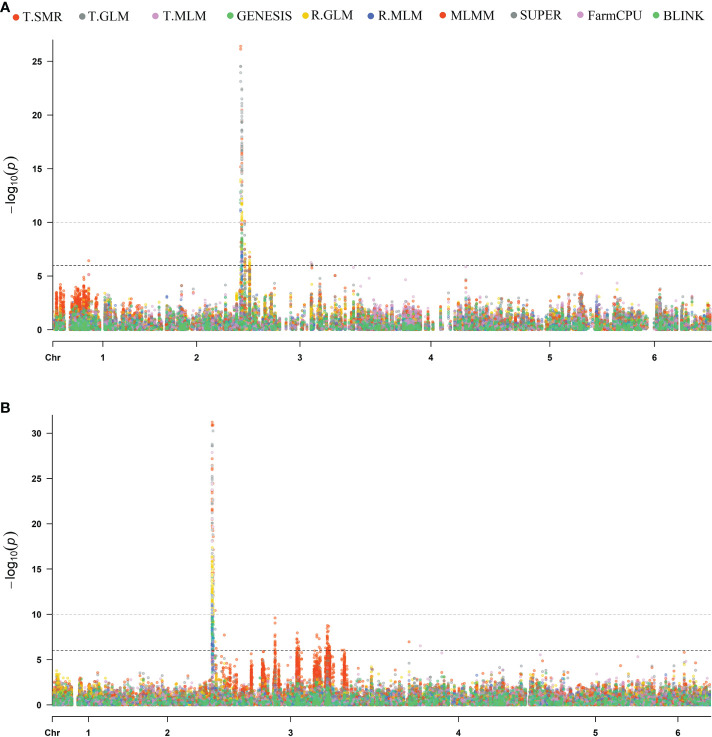
Manhattan plots of resistance to *P. effusa* race 5 in population segregating from Viroflay and Whale. GWAS were run with single marker regression (T.SMR), general linear model (T.GLM), mixed linear model (T.MLM), logistic mixed model (LMM) in GENESIS, and different GAPIT models as GLM, MLM, MLMM, SUPER, FarmCPU, and BLINK. The horizontal and vertical axis represents the genomic position of the SNP and association power for each SNP with the trait expressed as -log_10_(*P*-value). GWAS analysis was performed with SNPs called using the Monoe-Viroflay assembly **(A)** and Sp75 assembly **(B)**.

**Figure 4:**
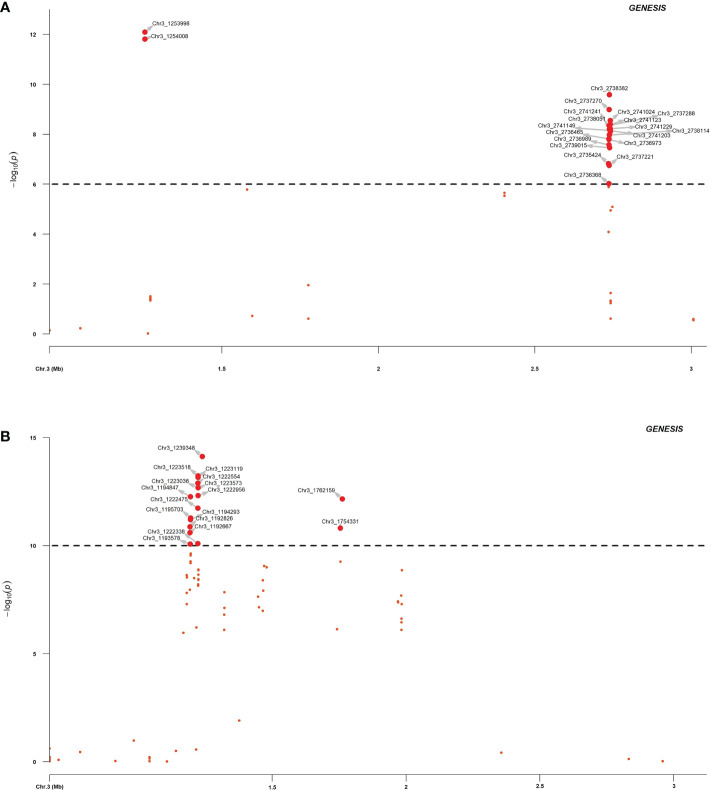
Regional association plot of *RPF3* resistance loci in spinach chromosome 3 between 0.5 to 3.0 Mb. The spinach breeding population segregating from a cross of Viroflay and Whale was inoculated with race 5 of *P. effusa* and was used for association analysis. The horizontal and vertical axis represents the genomic position of the SNP and association power for each SNP with the trait expressed as -log_10_(*P*-value). GWAS analysis was performed with SNPs called using the Monoe-Viroflay assembly **(A)** and Sp75 assembly **(B)**.

Association analysis was initially conducted to map the *RPF3* loci using 8,189 SNPs generated *via* whole genome shallow resequencing of a panel of 192 spinach lines based on the Monoe-Viroflay assembly. Different models were run on three GWAS programs to determine consistent associations. Several markers with a LOD value > 6.0 were identified across the tested models (**Figure 3A**) and the QQ plots show a wide divergence of observed *P*-values compared to that of expected *P*-values (**Supplementary Figure 2**). TASSEL program detected 35 SNP markers associated with LOD > 6.0 in the SMR model, 34 in the GLM model, and 16 in the MLM model. A total of 37 SNP markers were associated with LOD > 6 in one of the three models in the TASSEL program. Next, association analysis was performed in the GAPIT3 program and identified 38 SNPs in GLM, 13 in MLM, 2 in MLMM, 29 in SUPER, 3 in FarmCPU, and 1 in BLINK models. There were 38 SNPs associated with LOD > 6.0 in at least one of the GAPIT models. Finally, a logistic mixed model in the GENESIS R package that uses the in-built genetic relatedness matrix and principal components identified 20 SNP significantly associated markers with LOD > 6.0. All 20 SNP markers identified in the GENESIS program were detected by SMR and GLM models in TASSEL and at least in GLM and SUPER models in GAPIT. Of the total significant SNPs identified by different models and programs, 42 SNP showed LOD of > 6.0 in at least one of the three programs. Similarly, 20 SNPs showed substantial significance with LOD > 6 in GENESIS, plus two or more models of TASSEL and GAPIT, showing consistency, and are reported in detail as *RPF3* locus-associated SNPs (**Table 1** and **Figure 4A**). Of these 20 significant SNPs, two SNPs, Chr3_1253998 and Chr3_1254008, are localized in the 1.25 Mb region of chromosome 3, and all others remained in the 2.73-2.74 Mb region of Chromosome 3 (**Figure 4A**). The phenotypic variance (R^2^) explained by the SNP loci associated with resistance to downy mildew pathogen in the SMR model ranged from a minimum of 0.30 for Chr3_2737221 and was 0.57 for Chr3_1253998 and Chr3_125400820. The GENESIS model showed phenotypic variance explained by these 20 markers in the range of 0.19-0.40 and from 0.23-0.35 in the GAPIT GLM models (**Table 1**).

**Table 1:** List of SNP markers significantly associated with *P. effusa* race 5 resistance in spinach population segregating for *RPF3* resistance from Whale.

SNP	Chr	Position	Alleles (Ref/ Alt)	MAF	LOD (-log_10_ *P*) value											R^2^		
				TASSEL			GENESIS	GAPIT						Mean LOD value				
				SMR	GLM	MLM	LMM	GLM.R	MLM.R	MLMM	SUPER	FarmCPU	BLINK		SMR	GLM.R	GENESIS
*GWAS analysis using SNP markers called upon Monoe-Viroflay assembly*																		
Chr3_1253998	3	1253998	G/T	0.45	26.39	23.93	12.73	12.08	13.96	11.18	15.17	24.52	9.99	12.82	16.28	56.75	35.31	39.98
Chr3_1254008	3	1254008	G/T	0.45	26.14	23.12	12.72	11.81	13.76	11.03	0.01	24.52	0.41	0.20	12.37	56.65	34.69	39.00
Chr3_2735424	3	2735424	T/A	0.26	15.96	15.83	5.47	6.82	10.52	4.99	1.40	19.56	1.30	0.62	8.25	38.95	25.01	21.53
Chr3_2736368	3	2736368	C/T	0.27	13.84	13.40	5.11	6.02	9.78	4.01	0.87	18.61	0.45	0.31	7.24	34.07	22.92	18.74
Chr3_2736465	3	2736465	T/C	0.26	16.89	16.03	6.49	7.82	10.97	5.59	1.61	19.86	1.22	0.85	8.73	38.13	26.32	25.01
Chr3_2736973	3	2736973	G/A	0.24	15.49	15.85	6.24	7.79	10.43	4.90	0.86	19.36	0.63	0.23	8.18	35.62	24.76	24.90
Chr3_2736989	3	2736989	T/C	0.25	14.62	15.27	5.83	7.57	10.28	4.63	0.90	19.32	0.24	0.31	7.90	33.84	24.34	24.13
Chr3_2737221	3	2737221	A/G	0.24	12.86	13.49	5.68	6.75	10.17	5.45	1.21	18.57	0.43	0.32	7.49	30.00	24.02	21.26
Chr3_2737270	3	2737270	G/A	0.32	19.40	20.37	7.84	8.99	11.71	6.70	2.69	14.80	0.98	0.98	9.45	46.70	28.46	29.08
Chr3_2737288	3	2737288	T/C	0.23	16.94	17.04	7.32	8.35	11.72	6.95	2.30	20.18	1.34	0.97	9.31	37.68	28.52	26.86
Chr3_2738051	3	2738051	T/G	0.30	17.91	17.95	7.05	8.20	11.63	6.48	2.26	14.79	1.79	0.59	8.86	41.47	28.23	26.31
Chr3_2738114	3	2738114	G/A	0.24	16.58	16.72	6.54	7.97	12.12	6.90	2.21	22.43	1.02	0.80	9.33	36.52	29.68	25.52
Chr3_2738382	3	2738382	A/G	0.29	20.45	20.83	8.30	9.58	12.90	7.22	3.05	22.33	11.99	4.56	12.12	45.54	32.04	31.16
Chr3_2739015	3	2739015	G/A	0.24	17.77	17.23	6.49	7.45	12.14	6.40	1.91	21.49	2.87	0.59	9.43	40.61	29.75	23.73
Chr3_2741024	3	2741024	A/G	0.26	19.40	18.43	7.92	8.54	10.72	5.51	0.47	19.31	1.54	0.08	9.19	44.66	25.58	27.53
Chr3_2741123	3	2741123	C/G	0.23	16.18	16.65	7.20	8.40	11.91	6.87	0.73	15.92	3.72	4.66	9.22	35.16	29.08	27.01
Chr3_2741149	3	2741149	C/T	0.22	16.77	17.09	7.24	8.15	12.30	6.89	0.96	16.12	4.22	0.81	9.06	36.52	30.22	26.14
Chr3_2741203	3	2741203	A/G	0.22	16.32	16.82	6.73	8.14	11.82	6.40	0.50	15.30	3.49	0.98	8.65	36.06	28.79	26.12
Chr3_2741229	3	2741229	G/A	0.22	16.46	16.65	7.10	8.22	11.71	6.45	0.06	15.07	3.50	0.77	8.60	36.31	28.48	26.41
Chr3_2741241	3	2741241	G/T	0.22	17.17	17.41	7.31	8.10	11.89	6.75	13.76	21.08	3.47	1.06	10.80	38.26	29.00	25.98
*GWAS analysis using SNP markers called upon Sp75 assembly*																		
Chr3_1192667	3	1192667	A/T	0.38	27.18	25.24	16.13	10.60	14.45	8.86	4.91	1.94	1.70	1.34	11.23	0.59	35.67	0.42
Chr3_1192826	3	1192826	C/G	0.41	26.12	24.85	24.41	10.87	14.78	8.91	0.29	1.52	2.05	0.41	11.42	0.54	36.69	0.43
Chr3_1193578	3	1193578	T/C	0.41	21.38	20.08	10.12	10.07	13.83	8.25	0.31	0.91	1.88	0.17	8.70	0.46	33.79	0.40
Chr3_1194293	3	1194293	C/T	0.37	30.92	28.62	27.86	11.21	15.84	10.50	0.65	2.05	2.29	0.77	13.07	0.61	39.99	0.45
Chr3_1194847	3	1194847	T/G	0.40	31.21	28.78	18.05	12.26	16.37	11.14	1.88	1.32	2.67	5.36	12.90	0.60	41.71	0.49
Chr3_1195703	3	1195703	C/T	0.44	26.45	24.36	14.33	11.28	15.22	9.43	1.46	1.61	2.09	0.14	10.64	0.57	38.06	0.45
Chr3_1222338	3	1222338	A/C	0.46	16.37	16.72	7.59	10.09	12.93	6.74	1.13	0.61	0.64	0.12	7.29	0.36	31.10	0.40
Chr3_1222475	3	1222475	C/G	0.49	20.47	21.21	11.88	11.73	14.25	8.46	1.02	1.19	1.11	0.19	9.15	0.43	35.05	0.47
Chr3_1222554	3	1222554	C/A	0.49	23.66	25.04	13.90	12.89	15.54	9.70	2.60	2.18	2.10	3.56	11.12	0.49	39.04	0.52
*GWAS analysis using SNP markers called upon Sp75 assembly*					
Chr3_1222956	3	1222956	A/G	0.48	21.62	22.28	11.44	12.31	15.26	9.42	1.33	0.49	0.84	0.18	9.52	0.48	38.16	0.50
Chr3_1223036	3	1223036	C/T	0.49	23.65	24.52	14.25	12.68	15.38	9.83	1.75	0.76	0.74	0.08	10.36	0.50	38.54	0.51
Chr3_1223119	3	1223119	G/A	0.49	25.97	26.42	15.61	13.15	15.86	10.95	2.27	0.98	0.29	0.51	11.20	0.53	40.07	0.53
Chr3_1223518	3	1223518	A/T	0.49	23.40	23.75	14.12	13.22	16.28	10.90	2.11	0.61	0.13	1.10	10.56	0.51	41.41	0.54
Chr3_1223573	3	1223573	A/T	0.49	22.42	22.20	12.31	12.67	15.81	10.18	1.69	0.47	0.16	1.06	9.90	0.49	39.90	0.51
Chr3_1239348	3	1239348	T/C	0.49	30.83	28.59	23.74	14.12	17.36	12.08	7.84	1.08	20.51	5.67	16.18	0.63	44.92	0.57
Chr3_1754331	3	1754331	G/T	0.49	24.45	22.63	12.02	10.81	13.14	7.37	1.32	0.00	0.10	0.24	9.21	0.53	31.71	0.43
Chr3_1762159	3	1762159	T/C	0.44	30.90	30.24	19.66	12.16	15.08	9.74	5.97	0.49	6.79	5.54	13.66	0.61	37.61	0.49

SNP name defined as SNP position on the chromosome.

MAF is monor allele frequecy.

LOD (-LOG_10_P) value, with P value from the SMR, GLM, MLM models in TASSEL; logistic mixed model (LMM) in GENESIS; and GLM, MLM, MLMM, SUPER, FarmCPU, and BLINK models in GAPIT 3 R package.

Percentage of phenotypic variation (R^2^) explained by SNPs generated by TASSEL SMR model, GAPIT GLM model, and by GENESIS program.

In addition, GWAS analysis was performed with the 13,476 SNPs called on the same panel using the Sp75 genome assembly (**Figure 3B**). Seventeen SNPs were significantly associated with the *RPF3* locus with a LOD value greater than 10 in the GENESIS model and between 16 to 30 in the TASSEL GLM model (**Table 1** and **Figure 4B**). Of the 17 SNPs, 11 SNPs had a mean LOD value > 10.0 among the ten tested models. The 17 SNPs fall into five major regions at 1.19, 1.22, 1.23, 1.75, and 1.76 Mb of Sp75 chromosome 3. The phenotypic variance (R^2^) explained by these SNPs in the SMR model ranged from 0.36 for SNP marker Chr3_1222338 to 0.63 for SNP marker Chr3_1239348, while the R^2^ value for the same SNP was 0.40 and 0.57 in GENESIS model (**Table 1**).

### Candidate gene analysis

Twenty SNPs associated consistently in more than one model in each of the three GWAS programs were used to search for genes in the reference assembly, particularly to identify genes involved in disease resistance. The *P. effusa* race 5 resistance region in two regions of chromosome 3 mapped from the progeny population segregating from a cross of Viroflay and Whale, at 1.25 Mb and 2.73-2.74 Mb, harbor four genes within 20 Kb (**Table 2**). The 1.25 Mb region of the spinach chromosome 3 harbors genes SOV3g001240, SOV3g001250, and SOV3g001260 within 10 Kb of the peak SNPs. Gene SOV3g001250 is an NBS-LRR gene that encodes disease resistance protein and is 0.9 Kb from the two resistance-associated SNPs, Chr3_1253998 and Chr3_125400, in this study. The SOV3g001240 is annotated as an unknown protein and the SOV3g001260 is annotated as Protein transport protein Sec24-like. The other *RPF3* resistance-associated region at 2.73-2.74 Mb contains gene SOV3g002680 within 11.5 Kb, which is annotated as the Pectin acetylesterase gene. Furthermore, GWAS analysis and candidate search based on the Sp75 assembly found seventeen genes within 5, 10, and 20 Kb distance of the *RPF3*-associated SNPs (**Supplementary Table S1**). Of those, the SNPs in the 1.22-1.23 Mb region of Sp75 associated with *RPF3* resistance were within 2.41-3.65 Kb of the gene Spo12821 annotated as CC-NBS-LRR disease resistance protein. And the SNP Chr3_1239348 is located at 4.83 Kb of the gene Spo12919, which encodes *RAR1* protein, which is required for NBS-LRR protein accumulation and signaling in arabidopsis and contributes to resistance to plant pathogen (Cai et al., 2021).

### Genomic selection

**Figure 5:**
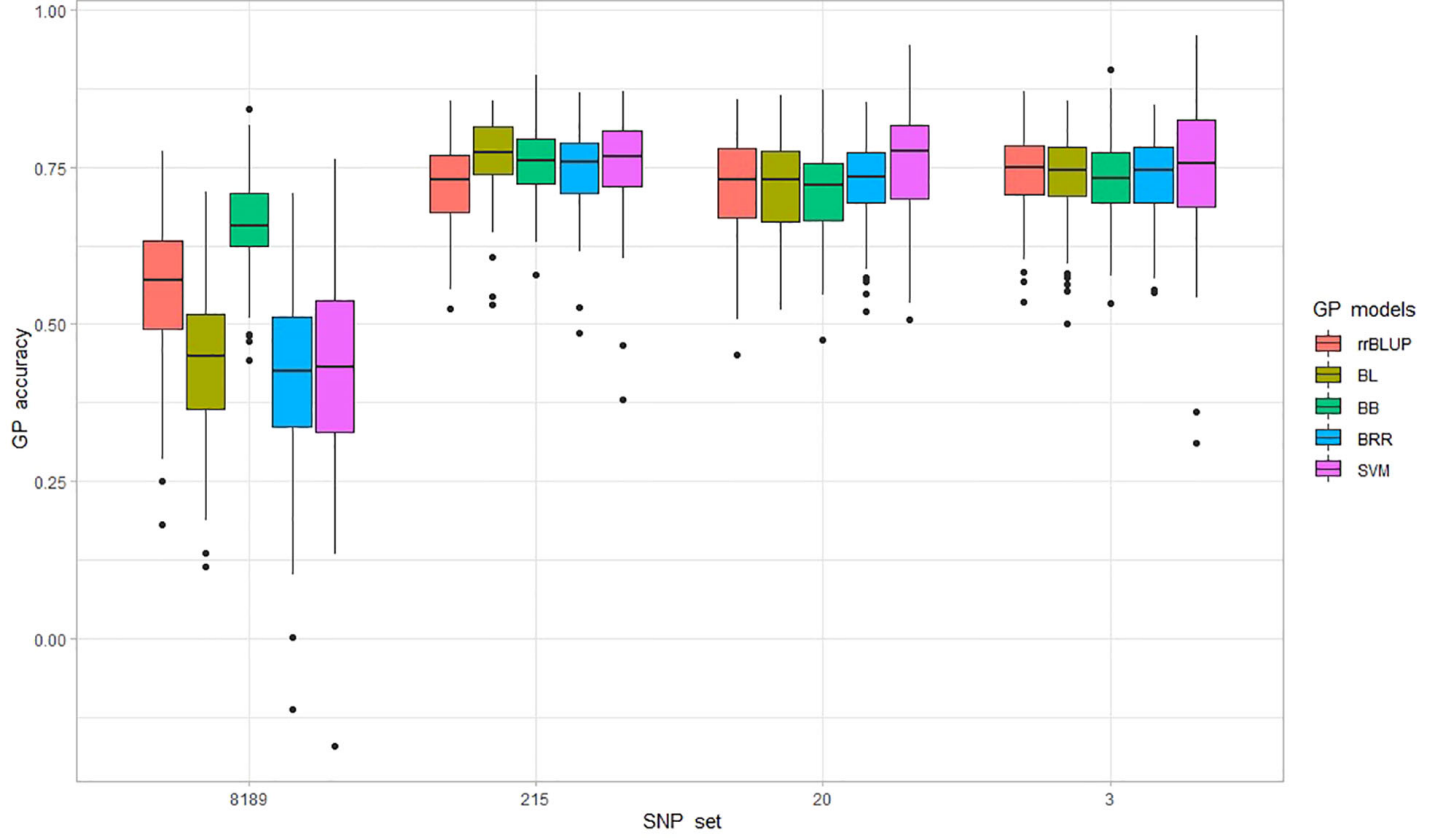
Prediction accuracy (PA) is measured as correlation (r-value) from 100 genomic predictions (GP) runs for *P. effusa* race 5 resistance. The population panels were evaluated for PA using five GP models, including rrBLUP, BL, BB, BRR, and SVM, and four marker sets comprising 8189, 215, 20, and 3 SNP markers.

The resistance to race 5 of *P. effusa* segregating from Whale and Viroflay was evaluated for prediction performance using a five-fold cross-validation approach (80% samples in the training population and the remaining 20% samples used for predicting breeding values) in five GS models. Average PA from 100 runs ranged from 0.42-0.66 among five different models in the full SNP dataset, with Bayesian B providing the highest prediction accuracy of 0.66 and the lowest standard deviation (**Figure 5** and **Table 3**). The other tested GP models, Bayesian LASSO, BRR, and SVM were similar in PA in a range of 0.42 to 0.43, while rrBLUP provided a PA of 0.56.

**Table 3:** Genomic prediction (r-value) was evaluated with five genomic predictions (GP) models for *P. effusa* race 5 resistance with four marker datasets.

GP model		rrBLUP	BL	BB	BRR	SVM	Average (All GP model)
8189	mean	0.56	0.43	0.66	0.42	0.43	0.50
	sd	0.12	0.12	0.08	0.14	0.15	
215	mean	0.72	0.77	0.76	0.75	0.75	0.75
	sd	0.07	0.06	0.06	0.07	0.08	
20	mean	0.72	0.72	0.72	0.73	0.76	0.73
	sd	0.08	0.07	0.07	0.07	0.09	
3	mean	0.74	0.73	0.73	0.73	0.75	0.74
	sd	0.07	0.07	0.07	0.07	0.11	
Average (SNP set)		0.69	0.66	0.71	0.66	0.67	0.68

Five different genomic selection (GS) models were evaluated for prediction accuracy using the complete SNP set comprising 8189 SNPs and three small sets of GWAS-associated SNPs.

In addition, prediction performance was tested in three smaller sets of markers comprising 215, 20, and 3 GWAS-associated SNPs to identify smaller sets of markers providing comparable PA to that of the entire marker set. The PA increased swiftly when a smaller number of GWAS-associated SNPs were used in all GP models; interestingly, a reduced number of GWAS-associated markers yielded higher PA as the three SNPs provided similar PA for all models obtained from 20 and 215 SNP markers. The average PA did not differ much across GS models when GP was performed using 215 SNPs (PA ranging between 0.72 to 0.77). Similar was the case with a minimum difference in mean PA across models with 20 SNP (PA in the range of 0.72 to 0.76) and 3 SNP sets (PA in the range of 0.73 to 0.75). It is important to note that the PA obtained using only three SNPs was equivalent to that of the 215 SNP set that provided the highest PA in this study. For the 3 SNP set, SVM was highest with PA of 0.75 ± 0.11, rrBLUP ranked second with PA of 0.74 ± 0.07, and the three Bayesian models ranked third with equal PA of 0.73 ± 0.07, making the SVM and rrBLUP the model of choice when in predicting downy mildew with a smaller number of markers. However, looking at the average PA of all four SNP sets, the Bayesian B model best predicted the downy mildew resistance in this population with a PA of 0.71, as other models averaged between 0.66-0.69. The PA obtained from three SNP markers appears to be a cost-effective approach and could facilitate predicting resistance to the *RPF3* locus.

## Discussion

Downy mildew is the major disease in commercial spinach production worldwide and in the United States, as the infected leaves are unmarketable. Spinach hybrid cultivars combining *RPF* alleles effective against multiple races of *P. effusa* from two parents are available (Correll et al., 2011; Bhattarai and Shi, 2021). However, new races of downy mildew pathogens are emerging and are overcoming the genetic resistance deployed in the new cultivars. Thus, there is an urgent need to investigate host-pathogen interaction by identifying and mapping more unique resistance sources and testing the functionality of the *RPF* genes to develop stable resistant cultivars against all known downy mildew races. Identifying tightly linked markers for each *RPF* locus against all races of *P. effusa* will enhance the efficiency and precision of molecular selection and expedite cultivar development. Cross population segregating for *RPF3* resistance locus from cultivar Whale was phenotyped for resistance against race 5 of *P. effusa* in this study. The F2 progenies generated from a cross between two inbreds, Whale and Viroflay, fit the 3:1 segregation ratio expected for traits governed by a dominant allele at a locus. Spinach is largely a dioecious crop with separate male and female plants, although some are monoecious (Morelock and Correll, 2007). The parent lines used in the crosses are often family pools of heterozygous genotypes making the linkage and QTL analysis more difficult in spinach (Bhattarai et al., 2020b). However, the GWAS approach allows us to map the trait in a mixed population or when there is a lack of fit of markers segregation for QTL mapping, as performed in this and previous spinach-downy mildew resistance mapping efforts (Bhattarai et al., 2020b; Bhattarai et al., 2021).

Low coverage sequencing (~1x), as pursued in this study to genotype the population panel, leads to high missing data (Malmberg et al., 2018). High missing data points are then imputed to infer missing genotype data based on haplotype information to increase marker density for downstream applications. The other important issue with low coverage sequencing is that heterozygotes genotypes are called homozygous, and such erroneous genotype calls primarily affect highly heterozygous species like spinach. We attempted to filter the imputed genotype calls by discarding the genotype call with a genotype probability (GP) value less than 0.90 to increase the accuracy of imputed genotype calls.

The phenotype and genotype data of the segregating population were used to map the resistance region by employing three GWAS programs to identify consistent sets of significant SNPs. Association analysis employed in multiple models in this study mapped the *RPF3* locus to a narrow region, in 1.25 Mb and 2.72 Mb of chromosome 3 (**Table 1**). Significant SNPs detected on multiple programs and association models were reported to associate with resistance to race 5 of *P. effusa*. The GENESIS logistic mixed model that incorporates models for binary phenotypes captured many SNPs detected by SMR and GLM in TASSEL and the GLM and SUPER models in GAPIT, making the association result more valid. Indeed, several SNP markers were associated in the two regions, increasing the confidence of this mapping result. This study employed ten GWAS models that reveal variable association results and a lack of constant hits across the models (**Table 1**), mainly when modeling for the qualitative or binary response variables. It is thus important to correctly evaluate the results from multiple GWAS models with varying functions and algorithms during GWAS analysis. The multi-locus GWAS models (MLMM, FarmCPU, and BLINK) narrowed to a few significant SNP hits but are missing other equally important markers and cannot be relied upon just based on these models. The SMR and GLM models in TASSEL and GAPIT programs identify more associated markers and appear to fit the downy mildew pathogen resistance phenotypes. The LMM model used in the GENESIS program identifies the markers also identified by the GLM models in TASSEL and GAPIT, making the GENESIS LMM model a better choice for GWAS analysis of qualitative disease phenotypes.

Resistance to downy mildew pathogen in spinach is hypothesized to be governed mainly by a major gene with a substantial effect on phenotype. The R^2^ values for the 20 SNP markers associated with the race 5 resistance averaged 40% and were 57% for the two SNPs at 1.25 Mb. This high R^2^ value, as expected for major locus, suggests the effectiveness of trait control. These results provide SNP markers explaining high phenotypic variance and close to the disease resistance candidate genes that will help in the efficient and effective deployment of the favorable resistance alleles. The GWAS analysis performed using the SNP marker dataset based on Sp75 assembly identified 17 associated SNPs in 1.19, 1.22, 1.23, 1.75, and 1.76 Mb of chromosome 3. GWAS performed with Monoe-Viroflay assembly maps the *RPF3* locus to the 1.25 Mb region coinciding with the major GWAS associated regions based on the Sp75 assembly at 1.19, 1.22, and 1.23 Mb. However, the 2.72 Mb region associated with the *RPF3* locus with the Monoe-Viroflay assembly was not observed with the Sp75 assembly. Instead, the Sp75 showed a second major QTL for the *RPF3* locus at 1.75-1.76 Mb making these two regions (2.72 Mb on Monoe-Viroflay and 1.75-1.76 Mb on Sp75) unique to associate with the *RPF3* resistance locus.

Association analysis in this study mapped the *RPF* resistance loci against downy mildew pathogen in spinach in the same region as previous studies (Feng et al., 2018a; Bhattarai et al., 2020b; Bhattarai et al., 2021). The *RPF1* locus segregating in progeny population from multiple parental crosses inoculated with *P. effusa* race 13 was mapped to 0.32-0.47, 0.69, 0.94-0.98, and 1.19-1.26 Mb region of chromosome 3 in Sp75 assembly (Bhattarai et al., 2020b). The *RPF3* locus from Whale segregating for *P. effusa* race 16 was mapped using GBS markers to a 0.57 Mb interval of chromosome 3 of the Sp75 assembly in the 0.65, 0.69, 1.10, and 1.22-1.23 Mb region (Bhattarai et al., 2021). This study further confined the *RPF3* locus to a narrow region between 1.22-1.23 Mb of Sp75 assembly. The GWAS analysis of resistance to downy mildew pathogen under natural inoculum pressure in the field also identified resistance-associated SNP markers at 0.94, 1.06, and 1.16 Mb regions of chromosome 3 (Bhattarai et al., 2022), supporting the presence of downy mildew QTLs in the region even for the field tolerance.

The high-confident SNPs identified from the GWAS analyses employing ten models (3 in TASSEL, 6 in GAPIT3, and 1 in GENESIS) were explored for the presence of disease resistance candidate genes near the associated regions (**Table 2**). The proximal end of chromosome 3 contains several other annotated disease resistance genes, including six NBS-LRR genes within 0.6-1.3 Mb and the markers for the *RPF1*, *RPF2*, and *RPF3* fall in the same region (Irish et al., 2008; Feng et al., 2018a; Bhattarai et al., 2020b; Bhattarai et al., 2021). This study identified a major SNP in the 1.25 Mb region of Chromosome 3 associated with *RPF3* loci lying near the disease resistance candidate gene SOV3g001250 contributing 57% of the phenotypic variance. The SOV3g001250 gene was reported as the potential candidate gene contributing to downy mildew resistance following GWAS analysis in a panel of more than 300 wild and cultivated accessions (Cai et al., 2021). Similarly, GWAS analysis with the SNPs based on Sp75 assembly mapped the *RPF3* locus mainly on the 1.22-1.23 Mb region of Chromosome 3 was 2.41-3.65 Kb of the gene Spo12821 annotated to encode CC-NBS-LRR disease resistance protein. Another major QTL located at 2.73 to 2.74 Mb of Chromosome 3 was 11.5 Kb of the Pectin acetylesterase gene, and this unique region showing association with resistance to *P. effusa* will be a new target to look for their functions in providing resistance to downy mildew pathogen. The NBS-LRR is the most common plant disease resistance gene that acts as a receptor of pathogen effectors to activate the signaling cascades for defense (Jones and Dangl, 2006), and these genes reported here are now the targets for validation and functional studies of downy mildew resistance *via* gene knockout and gene-expression experiments. The spinach downy mildew resistant locus *RPF1* through *RPF6* has been established and is being sought to characterize at the genetic and functional level. Efforts have been made to discover and describe the major and minor downy mildew resistance genes to combat the rapidly evolving new virulent races. Detailed genetic characterization of the resistance genes opens options to use molecular markers to select superior genotypes with an increased selection efficiency in terms of time and precision. Integrating molecular markers to deploy the resistant alleles during cultivar development are expected to shorten the breeding cycle. On the other hand, functional characterization of the R genes elucidates the genetic and operating mechanism of host-pathogen interaction, disease establishment, and pathogen strategies to overcome the available resistances. Such an advanced understanding of host-pathogen interaction at the molecular level will help formulate new ways to add genetic resistance to cultivar development.

**Table 2:** List of genes and gene functions located within 20 Kb distance from 20 GWAS associated SNP markers.

GWAS associated SNP			Gene description (Monoe-Viroflay annotation)				Distance from gene (Kb)	
SNP	Chr	Position	Gene ID	Start	End	Annotation	Start	End
Chr3_1253998	3	1253998	SOV3g001240	1237095	1244412	Unknown protein	16.903	9.586
		1253998	SOV3g001250	1246993	1253083	putative disease resistance protein	7.005	0.915
		1253998	SOV3g001260	1253496	1272509	Protein transport protein Sec24-like	0.502	18.511
Chr3_1254008	3	1254008	SOV3g001240	1237095	1244412	Unknown protein	16.913	9.596
		1254008	SOV3g001250	1246993	1253083	putative disease resistance protein	7.015	0.925
		1254008	SOV3g001260	1253496	1272509	Protein transport protein Sec24-like	0.512	18.501
Chr3_2735424	3	2735424	SOV3g002680	2722152	2752759	Pectin acetylesterase	13.272	17.335
Chr3_2736368	3	2736368	SOV3g002680	2722152	2752759	Pectin acetylesterase	14.216	16.391
Chr3_2736465	3	2736465	SOV3g002680	2722152	2752759	Pectin acetylesterase	14.313	16.294
Chr3_2736973	3	2736973	SOV3g002680	2722152	2752759	Pectin acetylesterase	14.821	15.786
Chr3_2736989	3	2736989	SOV3g002680	2722152	2752759	Pectin acetylesterase	14.837	15.77
Chr3_2737221	3	2737221	SOV3g002680	2722152	2752759	Pectin acetylesterase	15.069	15.538
Chr3_2737270	3	2737270	SOV3g002680	2722152	2752759	Pectin acetylesterase	15.118	15.489
Chr3_2737288	3	2737288	SOV3g002680	2722152	2752759	Pectin acetylesterase	15.136	15.471
Chr3_2738051	3	2738051	SOV3g002680	2722152	2752759	Pectin acetylesterase	15.899	14.708
Chr3_2738114	3	2738114	SOV3g002680	2722152	2752759	Pectin acetylesterase	15.962	14.645
Chr3_2738382	3	2738382	SOV3g002680	2722152	2752759	Pectin acetylesterase	16.23	14.377
Chr3_2739015	3	2739015	SOV3g002680	2722152	2752759	Pectin acetylesterase	16.863	13.744
Chr3_2741024	3	2741024	SOV3g002680	2722152	2752759	Pectin acetylesterase	18.872	11.735
Chr3_2741123	3	2741123	SOV3g002680	2722152	2752759	Pectin acetylesterase	18.971	11.636
Chr3_2741149	3	2741149	SOV3g002680	2722152	2752759	Pectin acetylesterase	18.997	11.61
Chr3_2741203	3	2741203	SOV3g002680	2722152	2752759	Pectin acetylesterase	19.051	11.556
Chr3_2741229	3	2741229	SOV3g002680	2722152	2752759	Pectin acetylesterase	19.077	11.53
Chr3_2741241	3	2741241	SOV3g002680	2722152	2752759	Pectin acetylesterase	19.089	11.518

The objective was to compare multiple machine learning models and the influence of a different set of markers in the prediction performance of downy mildew race-specific resistance in spinach. GS has recently been evaluated in spinach for resistance to white rust (Shi et al., 2022), field evaluated downy mildew (Bhattarai et al., 2022), while some other phenotypes are being assessed for GP in spinach (Bhattarai and Shi, 2021). Five GP models involving parametric models (rrBLUP, BB, BL, and BRR) and nonparametric models (SVM) in four marker datasets provide a comparative advantage between models and marker sets. The GS prediction models have different assumptions to treat marker effects, so the PA differs based on the phenotype and genetic architecture of the trait; however, there was not much difference in PA among the GS models. The Bayesian B model showed consistently higher PA in all marker datasets. The rrBLUP was superior in PA to SVM, BL, and BRR in the full dataset, but the smaller dataset of 215, 20, and 3 markers showed little difference in PA among the models. Bayesian models are known to provide higher PA for traits controlled by a few major QTLs with large effects (Daetwyler et al., 2010). The rrBLUP considers equal variances of all markers and incorporates genetic relationships, and low PA was reported for some traits, including field resistance to downy mildew in a spinach germplasm panel (Shikha et al., 2017; Islam et al., 2020; Bhattarai et al., 2022). Contrarily, the PA of Bayesian models and rrBLUP were similar for resistance to race 5 of downy mildew with large-effect QTLs in this study. The GWAS-associated SNP set showed improved PA for all models compared to the full dataset. PA of the full set of 8189 SNP was lower than the smaller GWAS-associated sets, which may be due to the overfitting of the GS model for a large number of SNPs in the full set, which has been reported for resistance to downy mildew and white rust resistance in spinach (Bhattarai et al., 2022; Shi et al., 2022) and stripe rust resistance in wheat (Merrick et al., 2021). There was minimal difference in PA when 3 or 20 GWAS-associated markers, notably three markers providing equal or higher PA for most of the tested models (increase by 0.02 for rrBlup, 0.01 for BL, BB, equal for BRR, and decrease by 0.02 for SVM). The 20 SNP markers lie in two *RPF* loci-associated regions but expand to short genomic regions and are in high LD, and this is why the three SNP markers provided equal or higher PA than the 20 markers. A relatively small number of GWAS-associated markers estimated comparable prediction to that of a larger SNP set providing an optimized marker set for no reduction in predictive ability. Equivalent PA obtained from a small SNP panel could attract adoption as it minimizes the cost of genotyping and favors using a small number of GWAS-associated SNP in GS. A commercial cultivar containing multiple resistant genes is an attractive option for the spinach industry as the cultivar will probably be durably resistant. Overall, this study showed the potential of accurately predicting and implementing GS for a major gene using nonparametric and parametric machine learning models, which may benefit from increased accuracy by including additional traits for selection.

## Conclusion

The *RPF3* resistance loci in the cultivar Whale have transmitted in a 3:1 ratio fitting the expected Mendelian segregation for a trait controlled by a major dominant locus. The *RPF3* resistance regions were mapped to the 1.25 Mb and 2.73-2.74 Mb of Monoe-Viroflay chromosomes 3 based on the significant and consistent association of 20 SNP markers across three GWAS programs and ten models. The *RPF3* locus was mapped to 1.19, 1.22, 1.23, 1.75, and 1.76 Mb of Sp75 chromosome 3. In this study, the 1.25 Mb associated with the *RPF3* locus from cultivar Whale is 0.9 Kb from the gene SOV3g001250, an NBS-LRR gene that encodes disease resistance protein. The *RPF3* locus in the 1.22-1.23 Mb region of Sp75 chromosome 3 is 2.41-3.65 Kb from the gene Spo12821 annotated as CC-NBS-LRR disease resistance protein. These genes can be targeted for functional tests of the *RPF3* locus to regulate resistance to downy mildew pathogens. This report further presented the GS tools with the ability to use three SNP markers to predict the *RPF3* resistance locus in spinach. Implementing GS will be a practical and attractive option with low-cost genotyping resources, incorporating other important traits, and simultaneous prediction of multi-trait data may provide increased PA. Continuous studies on genetics and molecular aspects of qualitative and quantitative host resistance, the evolution of pathogen races and the specificity of virulence factors, and the molecular mechanism and dynamics of host-pathogen interactions can provide innovative options to improve the development of downy mildew resistance cultivars.

## Data availability statement

The datasets generated by this study are available in FigShare: https://doi.org/10.6084/m9.figshare.20443017.

## Author contributions

AS and GB conceived the study. GB and DO maintain the downy mildew pathogen and performed phenotyping. JC managed the pathogen. GB performed sequencing and genotyping, planned and performed data analysis, and wrote the draft. AS, BM, and JC made edits, and GB made revisions. All authors contributed to the article and approved the submitted version.

## Funding

This research was supported by USDA-SCRI grant 2017-51181-26830, USDA-AMS SCMP grant 16SCCMAR0001, and USDA NIFA Hatch project ARK0VG2018 and ARK02440 to AS.

## Acknowledgments

The authors would like to acknowledge the funding support from USDA-AMS and USDA-SCRI, Genomics and Bioinformatics Service, Texas A&M for sequencing and genotyping. This research used the computational resources available through the Arkansas High Performance Computing Center at the University of Arkansas.

## Conflict of interest

The authors declare that the research was conducted in the absence of any commercial or financial relationships that could be construed as a potential conflict of interest.

## Publisher’s note

All claims expressed in this article are solely those of the authors and do not necessarily represent those of their affiliated organizations, or those of the publisher, the editors and the reviewers. Any product that may be evaluated in this article, or claim that may be made by its manufacturer, is not guaranteed or endorsed by the publisher.

## Supplementary material

Supplementary Figure 1 Genetic diversity of the spinach population segregating from a cross of cultivars Whale and Viroflay differentiated into two main sub-populations based on phylogenetic trees **(A)** drawn by neighbor-joining (NJ) method and the principal component analysis (PCA) plot **(B)** in GAPIT.

Supplementary Figure 2 QQ-plots of GWAS using different models in the TASSEL, GAPIT, and GENESIS programs using SNPs derived from the Monoe-Viroflay assembly. The horizontal and vertical axis represents the genomic position of the SNP and association power for each SNP with the trait expressed as -log_10_(*P*-value).

Supplementary Figure 3 QQ-plots of GWAS using different models in the TASSEL, GAPIT, and GENESIS programs using SNPs derived from the Sp75 assembly. The horizontal and vertical axis represents the genomic position of the SNP and association power for each SNP with the trait expressed as -log_10_(*P*-value).
