# Supplementary figures and images for: Mapping and selection of downy mildew resistance in spinach cv. whale by low coverage whole genome sequencing
Source: Front Plant Sci. 2022 Oct 6;13:1012923. doi: 10.3389/fpls.2022.1012923 (PMC9583407; doi:10.3389/fpls.2022.1012923)

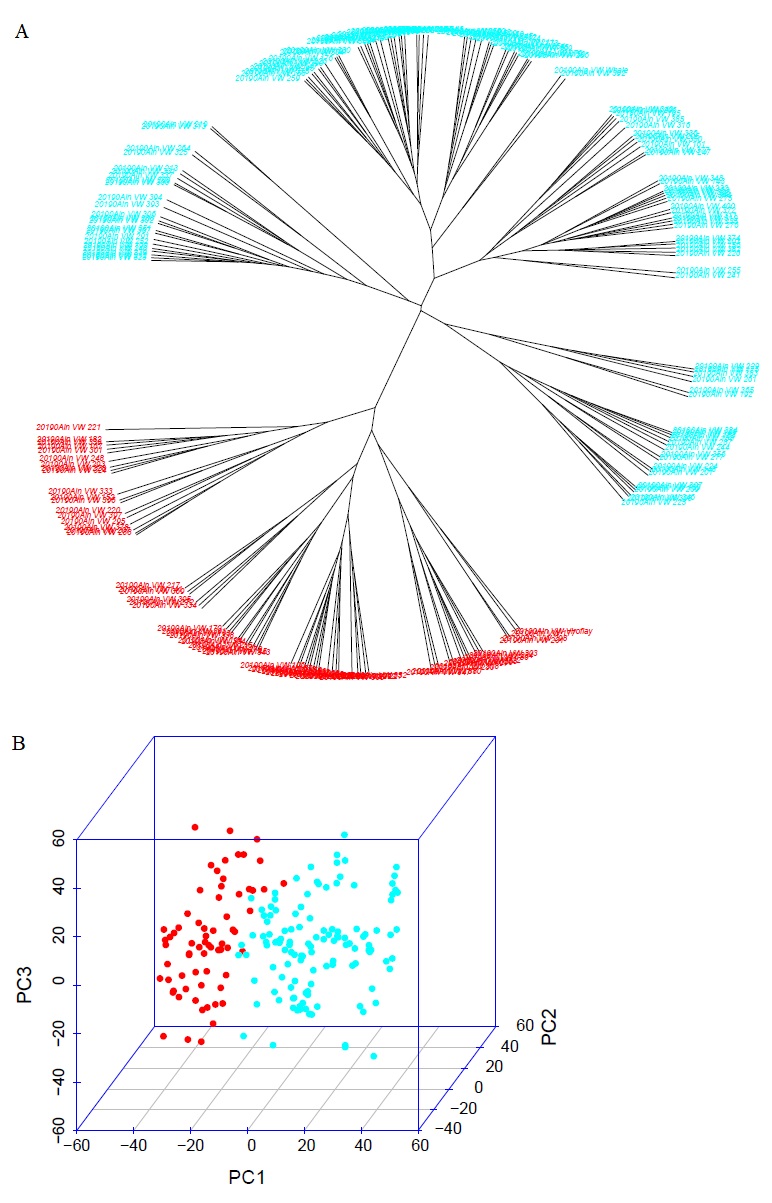

Supplement: Supplementary Figure 1 — Genetic diversity of the spinach population segregating from a cross of cultivars Whale and Viroflay differentiated into two main sub-populations based on phylogenetic trees (A) drawn by neighbor-joining (NJ) method and the principal component analysis (PCA) plot (B) in GAPIT. [file Image_1.jpeg]

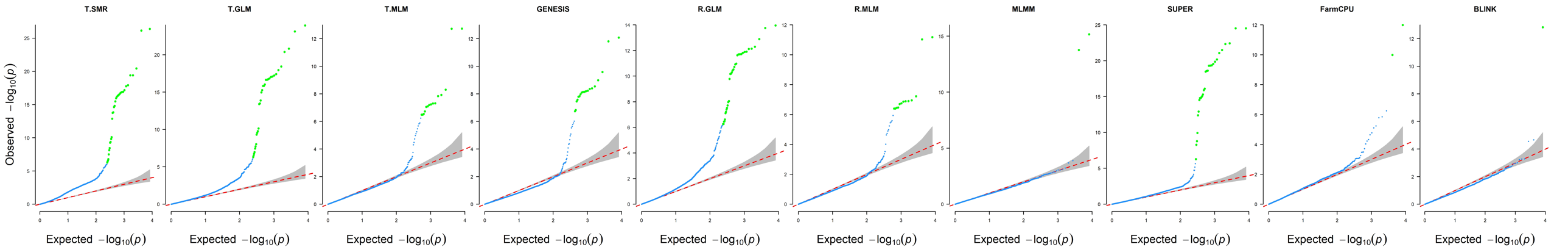

Supplement: Supplementary Figure 2 — QQ-plots of GWAS using different models in the TASSEL, GAPIT, and GENESIS programs using SNPs derived from the Monoe-Viroflay assembly. The horizontal and vertical axis represents the genomic position of the SNP and association power for each SNP with the trait expressed as -log10(P-value). [file Image_2.jpeg]

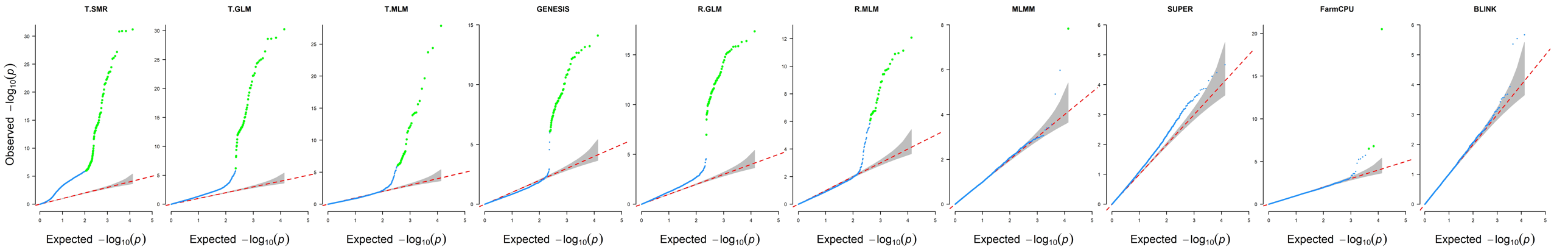

Supplement: Supplementary Figure 3 — QQ-plots of GWAS using different models in the TASSEL, GAPIT, and GENESIS programs using SNPs derived from the Sp75 assembly. The horizontal and vertical axis represents the genomic position of the SNP and association power for each SNP with the trait expressed as -log10(P-value). [file Image_3.jpeg]
